# Clinical Impact of RSV Vaccination in Hemodialysis Patients: Real-World Evidence on Hospitalization Risk and the Role of Chronic Lung Disease

**DOI:** 10.3390/arm94040045

**Published:** 2026-07-02

**Authors:** Francesca K. Martino, Francesca Fioretti, Lucia Federica Stefanelli, Gianni Carraro, Miriam Capuano, Giuseppe Scaparrotta, Federico Nalesso

**Affiliations:** Nephrology, Dialysis and Transplantation Unit, Department of Medicine (DIMED), University of Padova, 35128 Padua, Italy; francesca.fioretti.2@studenti.unipd.it (F.F.); luciafederica.stefanelli@unipd.it (L.F.S.); gianni.carraro_01@aopd.veneto.it (G.C.); miriam.capuano@studenti.unipd.it (M.C.); giuseppe.scaparrotta@aopd.veneto.it (G.S.); federico.nalesso@unipd.it (F.N.)

**Keywords:** respiratory syncytial virus vaccine, hemodialysis, chronic lung disease

## Abstract

**Highlights:**

**What are the main findings?**

- In hemodialysis patients, RSV vaccination was associated with a lower risk of hospitalization during respiratory infection episodes.
- In hemodialysis patients, chronic lung disease and a higher comorbidity index were associated with a significantly higher incidence of pneumonia, regardless of RSV vaccination status.

**What are the implications of the main findings?**

- RSV vaccination may reduce hospital care requirements during respiratory infections in hemodialysis patients.
- Hemodialysis patients with chronic lung disease and a high comorbidity index may require closer clinical surveillance for respiratory infections despite RSV vaccination.

**Abstract:**

**Background:** Respiratory syncytial virus (RSV) infection is a cause of respiratory morbidity in high-risk patients, including those with chronic lung disease (CLD) and those undergoing hemodialysis (HD). In HD patients, evidence on the clinical impact of RSV vaccination on respiratory complications remains limited. We aimed to assess the clinical impact of RSV vaccination in HD patients by comparing vaccinated and unvaccinated patients with a focus on CLD. **Methods:** We retrospectively evaluated 56 adult HD patients: 28 received the RSV vaccine in autumn 2024 and 28 did not. Clinical data were collected from electronic medical records. Outcomes included influenza-like illness (ILI), pneumonia, and respiratory infection requiring hospitalization between September 2024 and September 2025. **Results:** Patients had a mean age of 74.4 years and a median Charlson Comorbidity Index (CCI) of 10. The RSV-vaccinated group had a greater comorbidity burden than the unvaccinated group (CCI 11 IQR 10–12 vs. 9 IQR 8–11, *p* = 0.02) and a higher prevalence of CLD (46.4% vs. 25.0%, *p* = 0.09). During follow-up, 28 patients (50.0%) had at least one ILI episode, 23 (41.1%) developed pneumonia, and 15 (26.8%) were hospitalized for respiratory infection. The incidence of ILI was 46.4% in vaccinated patients and 53.6% in unvaccinated patients (*p* = 0.28), while the incidence of pneumonia was 39.3% and 42.9%, respectively (*p* = 0.78). Respiratory infection requiring hospitalization occurred in 14.3% of vaccinated patients and 39.3% of unvaccinated patients (*p* = 0.035). CLD was significantly associated with pneumonia (*p* = 0.001) and showed trends toward higher rates of ILI (*p* = 0.09) and hospitalization for respiratory infection (*p* = 0.1). **Conclusions:** In our exploratory study, RSV vaccination in HD patients was associated with fewer hospitalizations for respiratory infection, despite greater comorbidity in vaccinated patients. CLD was associated with a higher incidence of respiratory complications, particularly pneumonia. The retrospective design and small sample size do not allow definitive conclusions; future prospective studies with an adequate sample size are needed to confirm our results.

## 1. Introduction

Respiratory Syncytial Virus (RSV) infection represents an emerging concern [1] because of the high risk of complications and death in vulnerable populations [2], such as older adults and patients with chronic diseases [3,4,5,6,7,8]. In adults, RSV infection commonly causes upper respiratory tract symptoms, including rhinorrhea, cough, fever, and malaise [9,10,11]. In vulnerable individuals, however, it may progress to lower respiratory tract involvement, resulting in bronchiolitis, pneumonia, dyspnea, or exacerbation of chronic obstructive pulmonary disease (COPD) or heart failure [4,12,13,14]. In a phase 3 trial, vaccine efficacy remained high in participants with chronic lung disease (CLD), including COPD, asthma, interstitial lung disease, and chronic lung infection [3,10,15]. Furthermore, real-world studies have supported RSV vaccine effectiveness in reducing lower respiratory tract disease and hospitalization in high-risk older adults [16,17,18,19]. RSV infection is an important cause of respiratory morbidity and hospitalization, particularly among older adults and patients with relevant comorbidities. In a U.S. population-based study, hospitalization for community-acquired pneumonia occurred at an annual rate of 24.8 cases per 10,000 adults, with a marked increase in older age groups; RSV-specific pneumonia-related hospitalization was estimated at 0.7 cases per 10,000 adults per year [20]. In Italy, available data also suggest a heterogeneous RSV burden across clinical settings. A recent systematic review reported RSV seasonal attack rates ranging from 0.8‰ in community-dwelling older adults to 10.9% in hematological outpatients, with an overall RSV positivity of 4.5% among respiratory samples [21]. Importantly, among adults hospitalized with RSV infection, dialysis has been associated with a higher risk of mortality, with an odds ratio of approximately 4 [22]. In the Veneto region, where our center is located, RSV has been estimated to account for approximately 3.0–4.6% of respiratory infection-related hospitalizations among adults aged ≥65 years [23], although no local surveillance data are currently available specifically for hemodialysis (HD) patients.

HD patients may be at increased risk of RSV infection because of uremia-related impairment of both humoral and cellular immunity [24,25,26,27,28,29]. This risk is also influenced by chronic low-grade inflammation, older age, and the high prevalence of comorbidities in this population [30,31,32,33,34]. These factors may also affect the response to vaccination, although data on RSV vaccines in HD patients are scarce [29,35,36]. While RSV vaccination has shown benefit in older adults and other high-risk groups [28,35,37], its immunogenicity and effectiveness in HD patients have not been well defined. Only one study has evaluated RSV vaccine responses in dialysis patients, and no trial has enrolled HD patients exclusively [38]. Therefore, in the absence of specific evidence in HD patients, particularly those with CLD, current recommendations for dialysis patients are largely extrapolated from indirect evidence and from the effects of other vaccinations in this population [3].

After RSV vaccination became available in Italy in late 2023, our center offered it in autumn 2024 to a small number of HD patients judged to be at higher risk. To our knowledge, this is one of the first reports describing real-world outcomes after RSV vaccination in HD patients. Although the study is retrospective and limited in size, it may be useful because prospective data in this population are not yet available. We therefore compared vaccinated and unvaccinated HD patients during one year of follow-up, with a focus on respiratory outcomes and on whether chronic lung disease influenced both infection risk and the apparent benefit of vaccination.

## 2. Materials and Methods

We conducted a retrospective study at Padua University Hospital, Italy, to assess the clinical impact of RSV vaccination in HD patients, with particular attention to baseline pulmonary conditions. Vaccinated patients received a single intramuscular dose of Arexvy (RSVPreF3-AS01E, GSK), which contains a stabilized prefusion RSV F protein antigen combined with the AS01E adjuvant system. The antigen is derived from RSV subtype A, but the prefusion F protein is highly conserved across RSV subtypes and can induce immune responses against both RSV-A and RSV-B. They were compared with an age- and sex-matched control group of HD patients who had not received the RSV vaccine. The study was notified to the local Ethics Committee and conducted in accordance with the principles of the Declaration of Helsinki. All HD patients aged >18 years provided written informed consent to participate in the study.

According to the clinical records, the following variables were collected:

- demographic characteristics, including age, sex, dialysis vintage, and death during follow-up;
- the cause of end-stage kidney disease (ESKD);
- the presence and type of pulmonary disease, the need for oxygen support, and receipt of influenza and SARS-CoV-2 vaccination during 2024;
- the Charlson Comorbidity Index (CCI), adjusted for age, as a measure of comorbidity burden. The age-adjusted CCI was calculated by adding +1 point for patients aged 50–59 years, +2 for those aged 60–69 years, +3 for those aged 70–79 years, and +4 for those aged ≥80 years. The following comorbid conditions were included: diabetes, congestive heart failure, peripheral vascular disease, CLD, liver disease, hemiplegia, renal disease, hematologic malignancy or metastatic cancer, and acquired immunodeficiency syndrome. Notably, renal disease was considered with the same score in all patients;
- routine laboratory parameters, including hemoglobin, albumin, CRP, white blood cell count, uric acid, and urea. We considered the last blood samples collected before September 2024; in the vaccinated group, these were the last samples obtained before RSV vaccination;
- urine output and HD adequacy, assessed by Kt/V, measured during the month before RSV vaccination, and, for the unvaccinated group, during the equivalent period before September 2024, where K = dialyzer urea clearance (mL/min), t = duration of the dialysis session (minutes), and V = volume of distribution of urea;
- When available, nasopharyngeal swab results for RSV, SARS-CoV-2, and influenza were recorded during hospitalization episodes.

Outcomes: The impact of vaccination was assessed by evaluating respiratory clinical outcomes during the year after vaccination. We considered:

1. Influenza-like illness (ILI) was defined as fever or malaise associated with upper respiratory tract symptoms, such as rhinorrhea, cough, or sore throat. Cough was included according to standard epidemiological definitions [39]. Pneumonia was considered separately and required evidence of lower respiratory tract involvement.
2. Pneumonia, defined as clinical and/or radiological evidence of lower respiratory tract infection requiring antibiotic treatment.
3. Respiratory infection requiring hospitalization, defined as hospitalization during an episode of pneumonia or lower respiratory tract infection (LRTI).

**Control sampling:** Because an individual-matching strategy could not be applied, the control group was selected by random sampling from the overall HD cohort, with age- and sex-matched frequency. Patients were eligible for selection if they had not received RSV vaccination and were within the age range defined by the 5th–95th percentiles of the vaccinated group (63–88 years). From this eligible population, controls were randomly selected in order to reproduce, as closely as possible, the age and sex distribution of the vaccinated group.

**Sample size:** Given the lack of prior reports in the HD setting, no formal sample size calculation was performed. All HD patients who received RSV vaccination were enrolled. An equal number of unvaccinated HD patients were selected as controls using age- and sex-matched frequency sampling to minimize selection bias.

**Statistical analysis:** Continuous variables were reported as mean ± standard deviation (SD) or median with interquartile range (IQR), depending on their distribution, whereas categorical variables were reported as counts and percentages. The Kolmogorov–Smirnov test was used to assess the normality of continuous variables. The unpaired *t*-test or Mann–Whitney U-test was used to compare continuous variables, as appropriate. Categorical variables were compared using the chi-square test.

Univariate logistic regression was performed to explore the association between covariates and outcomes. Only outcomes with more than 10 events were considered eligible for univariate analysis. Given the small sample size, all regression analyses should be interpreted as exploratory. Continuous variables with non-normal distribution were log-transformed using the natural logarithm. All reported *p*-values were two-sided, and statistical significance was set at *p* < 0.05. Statistical analyses were performed using IBM SPSS Statistics, version 28.0.

## 3. Results

A total of 56 patients undergoing HD were enrolled, including 28 (50.0%) who had received RSV vaccination and 28 (50.0%) who had not. In 2024, 49 patients (87.5%) received influenza vaccination and 47 (83.9%) received SARS-CoV-2 vaccination. The main characteristics of the study population, overall and according to RSV vaccination status, are summarized in Table 1.

During the observation period, 15 deaths occurred in the overall HD cohort. No significant difference in all-cause mortality was observed between patients who did and did not receive RSV vaccination (*p* = 0.76) and among patients with and without CLD (*p* = 0.3).

### 3.1. Baseline Characteristics According to Chronic Lung Disease

Twenty patients (35.7%) had baseline CLD. Of these, 1 (1.8%) had chronic lung infection, 1 (1.8%) had occupational or environmental lung disease, 2 (3.6%) had interstitial or vascular lung disease, 2 (3.6%) had neoplastic lung disease, 5 (8.9%) had obstructive sleep apnea syndrome (OSAS), and 9 (16.1%) had obstructive airway disease.

Patients with CLD were generally similar to those without CLD in most baseline characteristics. However, influenza vaccination was less frequent in the CLD group than in the non-CLD group (75.0% vs. 94.4%, *p* = 0.035). Patients with CLD also showed a slightly higher Charlson Comorbidity Index, although this difference did not reach statistical significance (median 11 vs. 10, *p* = 0.12).

### 3.2. Incidence of Outcomes According to RSV Vaccination and Chronic Lung Disease

**Figure 1:**
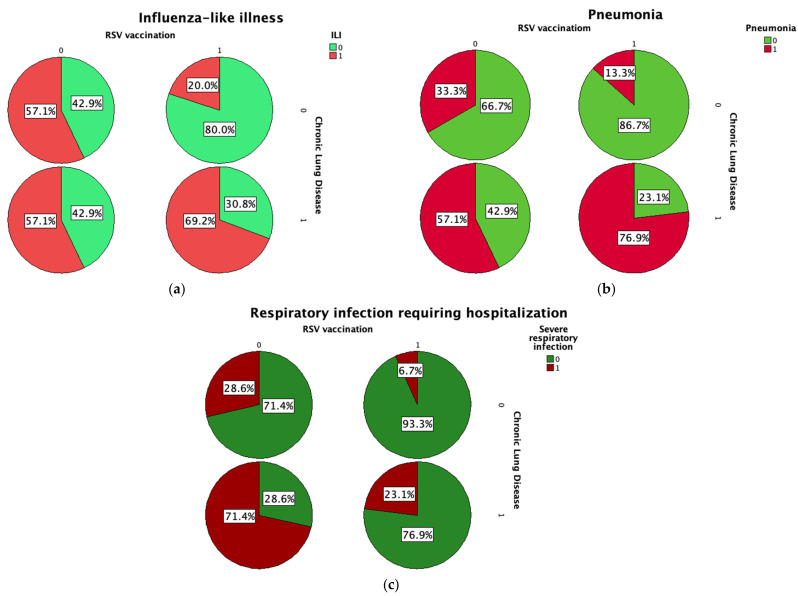
(**a**) Incidence of ILI according to RSV vaccination and chronic lung disease; (**b**) Incidence of pneumonia according to RSV vaccination and chronic lung disease. (**c**) Incidence of respiratory infection requiring hospitalization according to RSV vaccination and chronic lung disease.

During the 1-year follow-up, respiratory events were frequent among HD patients: 28 patients (50.0%) had at least one episode of ILI. 23 (41.1%) developed pneumonia, and 15 (26.8%) required hospitalization for respiratory infection. RSV vaccination was not associated with a significant reduction in ILI or pneumonia. However, vaccinated patients required hospitalization less frequently (14.3% vs. 39.3%, *p* = 0.035). Patients with CLD appeared to have a higher respiratory burden, with more pneumonia episodes (*p* = 0.001) and a non-significant trend toward more ILI and hospitalizations. Figure 1 shows the incidence of outcomes stratified by RSV vaccination status and CLD.

#### 3.2.1. Influenza-like Illness

No baseline variable was significantly associated with ILI in the univariate analysis. Baseline CLD and male sex showed only non-significant trends, with higher odds of ILI in patients with CLD and lower odds in male patients (OR 2.6, *p* = 0.098 and OR 0.36, *p* = 0.09, respectively; Table 2).

#### 3.2.2. Pneumonia

In the univariate analysis, baseline CLD was associated with a higher risk of pneumonia (OR 7.0, *p* = 0.002; Table 3). A higher Charlson Comorbidity Index was also associated with pneumonia (OR 22.03, *p* = 0.04; Table 3). RSV vaccination was not significantly associated with pneumonia risk (OR 0.86, *p* = 0.79; Table 3).

#### 3.2.3. Respiratory Infection Requiring Hospitalization

RSV vaccination was associated with a lower risk of hospitalization for respiratory infection in the univariate analysis (OR 0.26, *p* = 0.04). Baseline CLD showed a non-significant trend toward higher hospitalization risk (OR 2.76, *p* = 0.10; Table 4).

Among hospitalized patients, 14 underwent nasopharyngeal swab testing for RSV, SARS-CoV-2, and influenza at admission. Four patients tested positive for RSV (4/14, 28.6%); two of them had previously received RSV vaccination. One patient tested positive for influenza despite prior influenza vaccination, and one tested positive for SARS-CoV-2 despite prior SARS-CoV-2 vaccination.

## 4. Discussion

This study, to our knowledge, is the first to evaluate the association between RSV vaccination and respiratory outcomes in patients undergoing hemodialysis. Analyses were performed according to vaccination status and CLD status. RSV vaccination was not associated with a lower incidence of ILI or pneumonia; however, it was significantly associated with a reduction in respiratory infections requiring hospitalization. These findings suggest that RSV vaccination may mainly reduce severe outcomes rather than prevent all respiratory episodes. In the overall HD cohort, CLD and a higher CCI were significantly associated with pneumonia risk. Their relationship with respiratory infection requiring hospitalization was less clear, likely because of the small number of events.

### 4.1. Outcome Incidence

Respiratory events were common in our cohort. During follow-up, about 50% of patients had at least one ILI episode and about 40% developed pneumonia. The ILI rate was higher than the 24.8% reported in the Italian general population during the same period [40]. Other studies have similarly reported a higher incidence of pneumonia and related hospitalizations among elderly individuals and those with a high comorbidity burden [18,41,42]. Interpretation of the vaccination effect should take into account baseline differences between groups. RSV vaccination was preferentially offered to patients at higher clinical risk [43,44], and vaccinated patients had a higher CCI despite age- and sex-matching. This imbalance may have underestimated the protective effect of vaccination. In addition, the lower influenza vaccination rate among CLD patients may have contributed to their higher infection burden. Finally, the lack of systematic virological testing was mainly related to the retrospective design of our report. Only a minority of hospitalized patients were tested, with four RSV-positive cases, one influenza-positive case, and one SARS-CoV-2-positive case identified. Therefore, most respiratory events could not be assigned to a specific pathogen.

### 4.2. Clinical Impact of RSV Vaccination

RSV vaccination in hemodialysis patients was associated with a lower rate of hospitalization for respiratory infections, suggesting a possible role in attenuating severe complications rather than preventing infection altogether—a pattern consistent with real-world evidence from the general older adult population, where RSV vaccination has been shown to significantly reduce hospitalization rates [45].

The absence of a clear effect of RSV vaccination on ILI or pneumonia may partly reflect the immune dysfunction typical of HD patients. In this population, uremia can impair B-cell function, antibody production, and vaccine-induced humoral responses [46], which may limit protection against symptomatic infection [27,38,47,48]. By contrast, cellular immunity, although also affected, may be relatively better preserved and could help reduce progression to severe disease requiring hospitalization [25,30,49,50]. This pattern is consistent with what has been observed for other vaccines, such as SARS-CoV-2 and influenza vaccines, where protection against infection is generally less durable than protection against severe disease and hospitalization [48,49,51,52,53,54].

The higher CCI observed in the RSV-vaccinated group may have reduced the ability to detect a beneficial effect on ILI and pneumonia. In HD patients, vaccine response is likely influenced not only by age and comorbidity burden, but also by the underlying kidney disease and by previous or ongoing immunosuppressive therapy, variables that were not fully captured in our dataset [55,56]. This may be relevant because infection risk can differ across CKD etiologies: conditions such as ADPKD may carry a lower intrinsic risk [57,58,59] whereas others, including diabetic nephropathy [60], lupus nephritis [55], vasculitis [61], and hemolytic uremic syndrome [62], may be associated with systemic inflammation or immune dysfunction. Other factors, including the rate of CKD progression before dialysis [63,64,65], and previous solid organ transplantation may also affect the immune status at the time of vaccination [66]. These aspects should be assessed in future studies. Given the clinical heterogeneity of HD populations, validated tools to stratify the risk of severe RSV complications in this setting are needed, and larger multicenter cohorts would be required to develop and test a dedicated risk score.

The AS01E adjuvant used in Arexvy may be relevant in HD patients, given their impaired vaccine responses. Specifically, it enhances both humoral and cell-mediated immunity through activation of innate immune pathways [67,68]. In uremic patients, where antibody production and T-cell responses are often impaired [24,26,28], this adjuvant system may help strengthen vaccine-induced immunity. This suggests the need for future studies comparing adjuvanted and non-adjuvanted RSV vaccines also in HD patients. The durability of RSV vaccine protection in HD patients also remains uncertain. In solid organ transplant recipients, vaccine-induced antibody responses may decline rapidly because of chronic immunosuppression and immune senescence, often requiring repeat vaccination [38,66,69]. Similar mechanisms, including impaired B-cell function, reduced antibody production, and accelerated immune senescence, are also present in HD patients [25,26,31,38,47]. Whether a single RSV vaccine dose is sufficient in this setting, or whether booster doses may be needed, should be assessed in prospective immunogenicity studies.

### 4.3. Clinical Impact of Chronic Lung Disease

In the overall cohort, 20 patients (35.7%) had a history of CLD, of whom 5 (25%) required long-term oxygen therapy. CLD showed a consistent directional association across respiratory outcomes during the observation period and was a significant independent predictor of pneumonia (OR 7; *p* = 0.002). A statistically significant impact on ILI episodes or on respiratory infections requiring hospitalization was not demonstrated; however, effect estimates may reflect limited statistical power, although a true null effect cannot be excluded. These findings align with prior evidence establishing CLD as a major determinant of complications during acute viral respiratory illness [70,71,72,73].

The co-occurrence of CLD and ESRD in this cohort identifies a particularly high-risk subgroup in whom careful clinical surveillance and prioritization of RSV vaccination appear warranted. Whether this combination confers additive or synergistic susceptibility to severe RSV disease remains an open question that prospective studies with adequate sample sizes should address.

### 4.4. Limitations

This study has several limitations. First, it was retrospective and included a small number of patients, which limits the generalizability of the results. The use of electronic health records and regular nephrologist assessments improved data reliability, but some limitations of retrospective data collection remain. Nasopharyngeal swabs were not performed systematically, so the viral cause of most ILI and pneumonia episodes could not be defined. Even when virological testing is performed, however, a negative result does not completely exclude respiratory viral infection, since test sensitivity may be affected by pre-analytical factors such as timing and quality of sampling, viral load, and sample handling [74,75,76,77]. Therefore, the lack of systematic swabbing remains one of the main limitations of our report, and the effect of RSV vaccination on these outcomes should be interpreted with caution. The small sample size also limited subgroup analyses and reduced the statistical power to detect differences between covariates. The wide 95% confidence intervals reflect this limitation. Therefore, all regression analyses should be considered exploratory and need confirmation in larger cohorts. Based on our results, we estimated that future studies would require 42 patients per group to detect a significant difference in respiratory infection-related hospitalization, assuming 80% power and a two-sided alpha level of 0.05. Another important limitation is the baseline imbalance between groups. RSV vaccination was preferentially offered to patients considered at higher clinical risk, as expected in a real-world setting. As a result, vaccinated patients had a higher comorbidity burden, including more CLD and a higher CCI. In addition, lower influenza vaccination rates among CLD patients may have contributed to their higher infection burden. This point is clinically relevant, since chronic lung disease has a more direct impact on respiratory infection risk than kidney disease alone, and patients with CLD therefore have a particular need for complete vaccination coverage. These imbalances may have made it harder to detect a vaccine benefit. Therefore, the lower rate of respiratory infection-related hospitalization among vaccinated patients should be interpreted cautiously.

Despite these limitations, this study provides early real-world data on RSV vaccination in HD patients, a population for which evidence is still limited. The results are not sufficient to draw firm conclusions on vaccine effectiveness, but they may help generate hypotheses and support future prospective studies with systematic virological testing.

## 5. Conclusions

In our cohort of HD patients with a high comorbidity burden, RSV vaccination was not associated with a reduction in overall ILI or pneumonia incidence but was significantly associated with a lower rate of respiratory infections requiring hospitalization, suggesting a possible role in reducing severe complications. In our series, CLD was significantly associated with pneumonia in this cohort, identifying a subgroup of HD patients at particularly elevated respiratory risk for whom vaccination prioritization appears clinically justified. Given the small sample size and retrospective design, as well as lack of systematic nasopharyngeal swabs, these findings should be interpreted cautiously. Nevertheless, this study provides preliminary real-world evidence on RSV vaccination in HD patients and highlights the need for larger prospective studies to better define vaccine effectiveness and risk stratification in this high-risk population.

## Figures and Tables

**Table 1:** Main characteristics of the HD patients.

Variables	Whole Population (N = 56)	No RSV Vaccination (N = 28)	RSV Vaccination (N = 28)	*p*
Male	38 (67.9%)	19 (67.9%)	19 (67.9%)	0.999
Baseline CKD:				0.82
ADPKD	10 (17.9%)	4 (14.3%)	6 (21.4%)	
Glomerulonephritis	8 (14.3%)	4 (14.3%)	4 (14.3%)	
Nephropathy in systemic disease	8 (14.3%)	5 (17.9%)	3 (10.7%)	
Others or Unknown	30 (53.6%)	15 (53.6%)	15 (53.6%)	
CLD	20 (35.7%)	7 (25%)	13 (46.4%)	0.09
O2 support	5 (8.9%)	2 (7.1%)	3 (10.7%)	0.6
Influenza vaccination	49 (87.5%)	26 (92.9%)	23 (82.1%)	0.22
SARS-CoV-2 vaccination	47 (83.9%)	23 (82.1%)	24 (85.7%)	0.72
No urine output	29 (51.7%)	14 (50%)	15 (53.6%)	
Age (years)	74.4 ± 8.7	74 ± 9.9	74.8 ± 7.6	0.91
Dialysis vintage (months)	41 (24–76.5)	39 (22.25–52.25)	48 (24–102)	0.41
Charlson comorbidity index	10 (9–11.75)	9 (8–11)	11 (10–12)	0.02
CRP (mg/L)	1.5 (0.9–4.5)	1.8 (1.1–4.9)	1.36 (0.75–2.5)	0.12
Hemoglobin (g/L)	106.6 ± 12.1	105.3 ± 11.6	107.8 ± 12.7	0.5
Albumin (g/L)	35 (33–38.7)	34 (32.25–37.25)	36.5 (34–39)	0.2
KT/V	1.2 ± 0.18	1.26 ±0.25	1.2 ± 0.18	0.83
BMI (Kg/m^2^)	25.8 ± 4.1	25.5 ± 2.9	26.2 ± 5.3	0.47

Footnotes: CKD = chronic kidney disease, ADPKD = Autosomal Dominant Polycystic Kidney Disease, CRP = C-Reactive Protein, KT/V = Single-pool Kt/V, BMI = body mass index.

**Table 2:** Influenza-like illness univariate analysis.

Covariates	OR	95% CI	*p*
Male	0.36	0.11–1.17	0.09
Baseline CLD	2.6	0.84–8.07	0.098
RSV vaccination	0.56	0.19–1.62	0.29
Influenza Vaccination	1.39	0.28–6.86	0.69
SARS-CoV-2 Vaccination	2.27	0.51–10.8	0.28
No urine output	1.45	0.5–4.2	0.49
Age	0.99	0.93–1.05	0.64
Dialysis vintage (*)	1.38	0.81–2.3	0.23
Charlson comorbidity index (*)	1.33	0.12–14.8	0.81
CRP (*)	1.52	0.82–2.8	0.18
Hemoglobin	0.99	0.95–1.04	0.81
Albumin (*)	8.1	0.19–34.4	0.28
KT/V	0.15	0.1–2.4	0.18
BMI	0.98	0.8–1.12	0.52

Footnotes: CLD = Chronic lung disease, CRP = C-Reactive Protein, KT/V = Single-pool Kt/V, BMI = body mass index. (*) Log transformed variable.

**Table 3:** Pneumonia univariate analysis.

Covariates	OR	95% CI	*p*
Male	1.23	0.39–3.8	0.72
Baseline CLD	7	2.07–23.6	0.002
RSV vaccination	0.86	0.3–2.5	0.79
Influenza Vaccination	4.3	0.78–24.5	0.19
SARS-CoV-2 Vaccination	3.5	0.78–15.9	0.1
No urine output	2.94	0.95–9.01	0.06
Age	1.06	0.99–1.14	0.093
Dialysis vintage (*)	1.18	0.69–1.98	0.53
Charlson comorbidity index (*)	22.03	1.17–41.4	0.04
CRP (*)	0.57	0.29–1.35	0.11
Hemoglobin	1.001	0.96–1.05	0.95
Albumin (*)	0.98	0.88–1.1	0.79
KT/V	2.11	0.15–29.1	0.58
BMI	0.9	0.78–1.04	0.16

Footnotes: CLD = Chronic lung disease, CRP = C-Reactive Protein, KT/V = Single-pool Kt/V, BMI = body mass index. (*) Log-transformed variable.

**Table 4:** Respiratory infection requiring hospitalization univariate analysis.

Covariates	OR	95% CI	*p*
Male	1.6	0.47–5.5	0.45
Baseline CLD	2.76	0.82–9.33	0.1
RSV vaccination	0.26	0.07–0.9	0.04
Influenza Vaccination	0.42	0.046–3.78	0.44
SARS-CoV-2 Vaccination	1.46	0.31–6.76	0.63
No urine output	2	0.58–6.9	0.27
Age	1.06	0.98–1.14	0.17
Dialysis vintage (*)	1.38	0.75–2.5	0.35
Charlson comorbidity index (*)	14.9	0.6–37.3	0.1
CRP (*)	0.68	0.33–1.42	0.31
Hemoglobin	0.99	0.94–1.04	0.57
Albumin (*)	0.25	0.01–9.7	0.56
KT/V	0.68	0.036–12.9	0.8
BMI	0.93	0.79–1.08	0.34

Footnotes: CLD = Chronic lung disease, CRP = C-Reactive Protein, KT/V = Single-pool Kt/V, BMI = body mass index. (*) Log transformed variable.

## Data Availability

Data are available on request due to privacy or ethical restrictions.

## Abbreviations

The following abbreviations are used in this manuscript:

RSV	Respiratory syncytial virus
COPD	Chronic obstructive pulmonary disease
CLD	Chronic lung disease
HD	Hemodialysis
CKD	Chronic kidney disease
ESKD	End-stage kidney disease
CCI	Charlson comorbidity index
ILI	Influenza-like illness
SD	Standard deviation
IQR	Interquartile range
